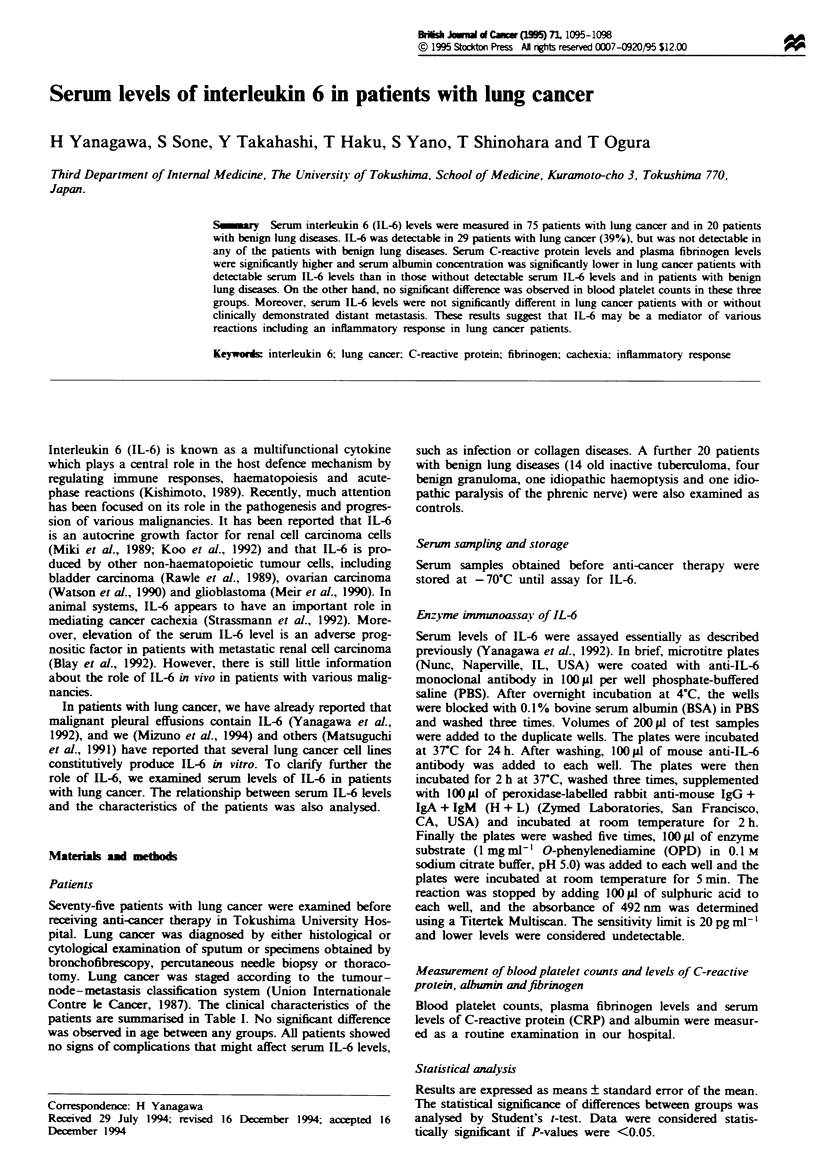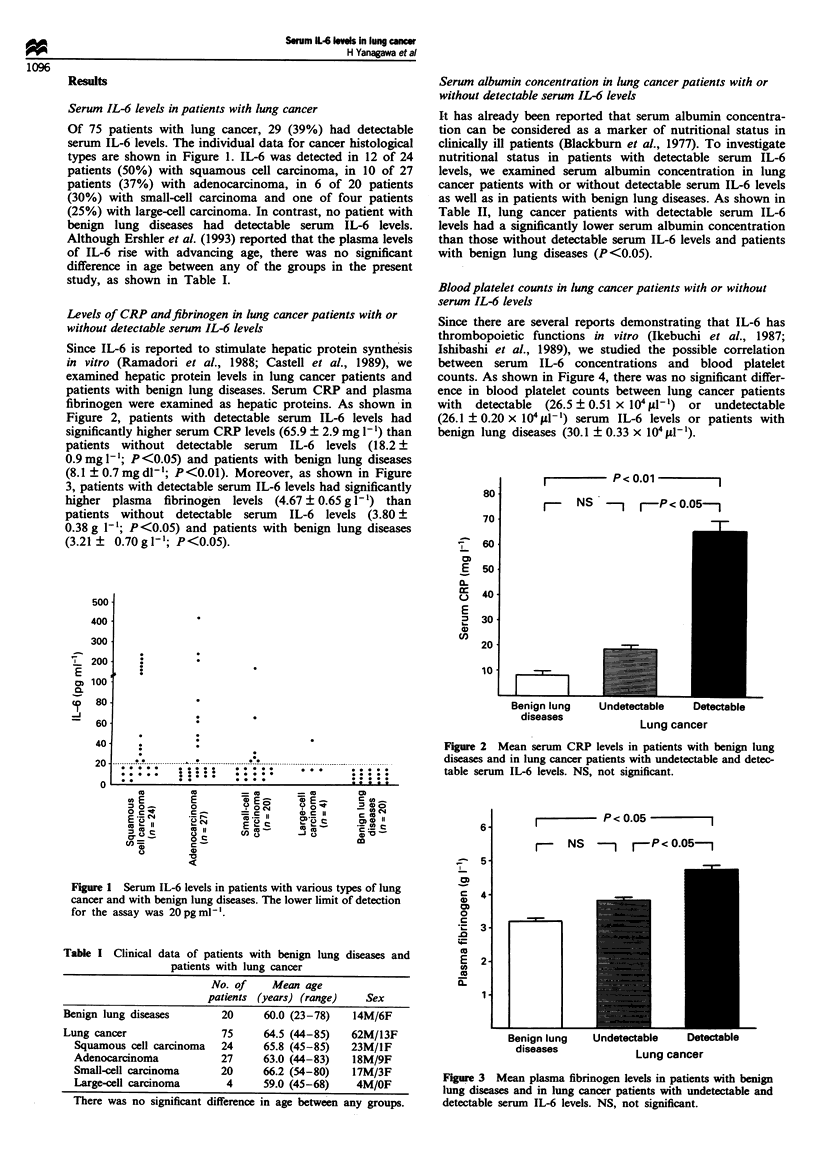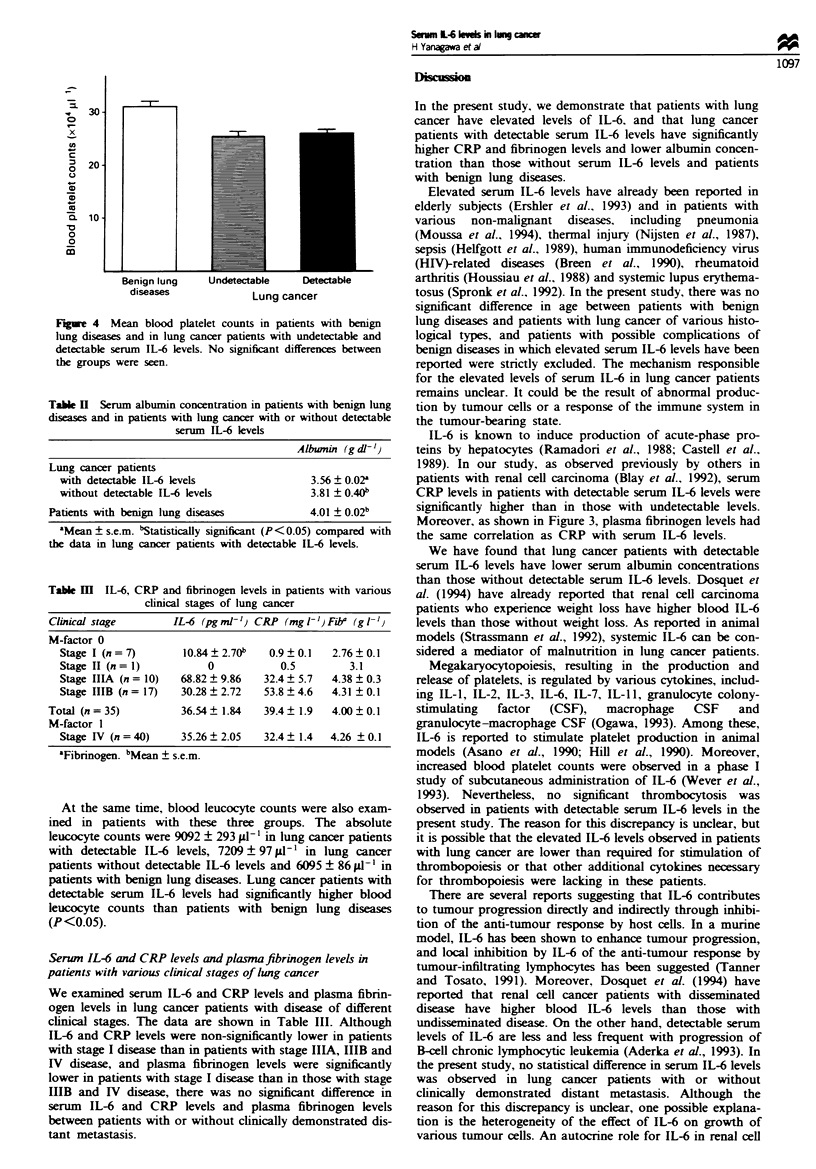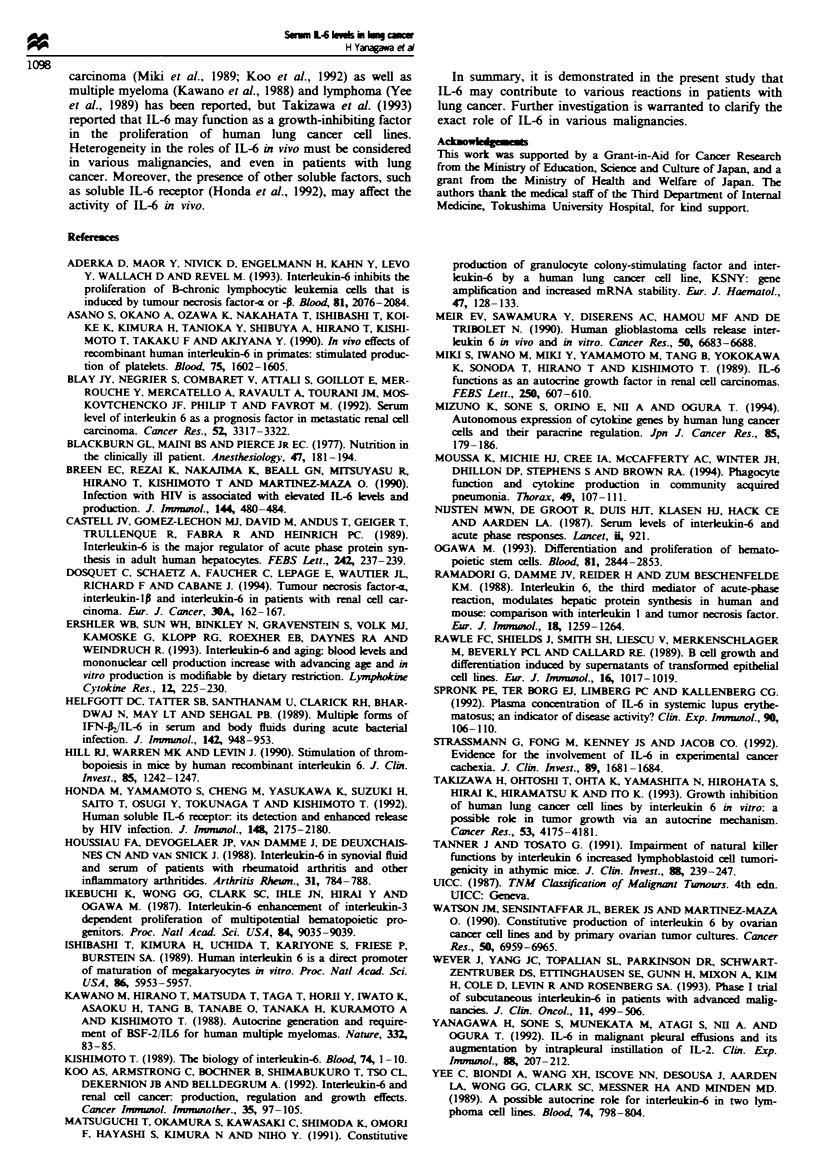# Serum levels of interleukin 6 in patients with lung cancer.

**DOI:** 10.1038/bjc.1995.212

**Published:** 1995-05

**Authors:** H. Yanagawa, S. Sone, Y. Takahashi, T. Haku, S. Yano, T. Shinohara, T. Ogura

**Affiliations:** Third Department of Internal Medicine, University of Tokushima, School of Medicine, Japan.

## Abstract

Serum interleukin 6 (IL-6) levels were measured in 75 patients with lung cancer and in 20 patients with benign lung diseases. IL-6 was detectable in 29 patients with lung cancer (39%), but was not detectable in any of the patients with benign lung diseases. Serum C-reactive protein levels and plasma fibrinogen levels were significantly higher and serum albumin concentration was significantly lower in lung cancer patients with detectable serum IL-6 levels than in those without detectable serum IL-6 levels and in patients with benign lung diseases. On the other hand, no significant difference was observed in blood platelet counts in these three groups. Moreover, serum IL-6 levels were not significantly different in lung cancer patients with or without clinically demonstrated distant metastasis. These results suggest that IL-6 may be a mediator of various reactions including an inflammatory response in lung cancer patients.


© 1995 Stockton Press All rights reserved 0007-0920/95 $12.00

### Serum levels of interleukin 6 in patients with lung cancer

H Yanagawa, S Sone, Y Takahashi, T Haku, S Yano, T Shinohara and T Ogura

*Third Department of Internal Medicine, The University of Tokushima, School of Medicine, Kuramoto-cho 3, Tokushima 770, Japan.*

**Summary** Serum interleukin 6 (IL-6) levels were measured in 75 patients with lung cancer and in 20 patients with benign lung diseases. IL-6 was detectable in 29 patients with lung cancer (39%), but was not detectable in any of the patients with benign lung diseases. Serum C-reactive protein levels and plasma fibrinogen levels were significantly higher and serum albumin concentration was significantly lower in lung cancer patients with detectable serum IL-6 levels than in those without detectable serum IL-6 levels and in patients with benign lung diseases. On the other hand, no significant difference was observed in blood platelet counts in these three groups. Moreover, serum IL-6 levels were not significantly different in lung cancer patients with or without clinically demonstrated distant metastasis. These results suggest that IL-6 may be a mediator of various reactions including an inflammatory response in lung cancer patients.

**Keywords:** interleukin 6; lung cancer; C-reactive protein; fibrinogen; cachexia; inflammatory response

Interleukin 6 (IL-6) is known as a multifunctional cytokine which plays a central role in the host defence mechanism by regulating immune responses, haematopoiesis and acute-phase reactions (Kishimoto, 1989). Recently, much attention has been focused on its role in the pathogenesis and progression of various malignancies. It has been reported that IL-6 is an autocrine growth factor for renal cell carcinoma cells (Miki *et al.*, 1989; Koo *et al.*, 1992) and that IL-6 is produced by other non-haematopoietic tumour cells, including bladder carcinoma (Rawle *et al.*, 1989), ovarian carcinoma (Watson *et al.*, 1990) and glioblastoma (Meir *et al.*, 1990). In animal systems, IL-6 appears to have an important role in mediating cancer cachexia (Strassmann *et al.*, 1992). Moreover, elevation of the serum IL-6 level is an adverse prognositic factor in patients with metastatic renal cell carcinoma (Blay *et al.*, 1992). However, there is still little information about the role of IL-6 *in vivo* in patients with various malignancies.

In patients with lung cancer, we have already reported that malignant pleural effusions contain IL-6 (Yanagawa *et al.*, 1992), and we (Mizuno *et al.*, 1994) and others (Matsuguchi *et al.*, 1991) have reported that several lung cancer cell lines constitutively produce IL-6 *in vitro*. To clarify further the role of IL-6, we examined serum levels of IL-6 in patients with lung cancer. The relationship between serum IL-6 levels and the characteristics of the patients was also analysed.

### Materials and methods

### *Patients*

Seventy-five patients with lung cancer were examined before receiving anti-cancer therapy in Tokushima University Hospital. Lung cancer was diagnosed by either histological or cytological examination of sputum or specimens obtained by bronchofibrescopy, percutaneous needle biopsy or thoracotomy. Lung cancer was staged according to the tumour–node–metastasis classification system (Union Internationale Contre le Cancer, 1987). The clinical characteristics of the patients are summarised in Table I. No significant difference was observed in age between any groups. All patients showed no signs of complications that might affect serum IL-6 levels, such as infection or collagen diseases. A further 20 patients with benign lung diseases (14 old inactive tuberculoma, four benign granuloma, one idiopathic haemoptysis and one idiopathic paralysis of the phrenic nerve) were also examined as controls.

### *Serum sampling and storage*

Serum samples obtained before anti-cancer therapy were stored at −70°C until assay for IL-6.

### *Enzyme immunoassay of IL-6*

Serum levels of IL-6 were assayed essentially as described previously (Yanagawa *et al.*, 1992). In brief, microtitre plates (Nunc, Naperville, IL, USA) were coated with anti-IL-6 monoclonal antibody in 100 µl per well phosphate-buffered saline (PBS). After overnight incubation at 4°C, the wells were blocked with 0.1% bovine serum albumin (BSA) in PBS and washed three times. Volumes of 200 µl of test samples were added to the duplicate wells. The plates were incubated at 37°C for 24 h. After washing, 100 µl of mouse anti-IL-6 antibody was added to each well. The plates were then incubated for 2 h at 37°C, washed three times, supplemented with 100 µl of peroxidase-labelled rabbit anti-mouse IgG + IgA + IgM (H + L) (Zymed Laboratories, San Francisco, CA, USA) and incubated at room temperature for 2 h. Finally the plates were washed five times, 100 µl of enzyme substrate (1 mg ml$^{-1}$ *O*-phenylenediamine (OPD) in 0.1 M sodium citrate buffer, pH 5.0) was added to each well and the plates were incubated at room temperature for 5 min. The reaction was stopped by adding 100 µl of sulphuric acid to each well, and the absorbance of 492 nm was determined using a Titertek Multiscan. The sensitivity limit is 20 pg ml$^{-1}$ and lower levels were considered undetectable.

### *Measurement of blood platelet counts and levels of C-reactive protein, albumin and fibrinogen*

Blood platelet counts, plasma fibrinogen levels and serum levels of C-reactive protein (CRP) and albumin were measured as a routine examination in our hospital.

### *Statistical analysis*

Results are expressed as means ± standard error of the mean. The statistical significance of differences between groups was analysed by Student's *t*-test. Data were considered statistically significant if *P*-values were $<0.05$.

Correspondence: H Yanagawa
Received 29 July 1994; revised 16 December 1994; accepted 16 December 1994

### Results

*Serum IL-6 levels in patients with lung cancer*

Of 75 patients with lung cancer, 29 (39%) had detectable serum IL-6 levels. The individual data for cancer histological types are shown in Figure 1. IL-6 was detected in 12 of 24 patients (50%) with squamous cell carcinoma, in 10 of 27 patients (37%) with adenocarcinoma, in 6 of 20 patients (30%) with small-cell carcinoma and one of four patients (25%) with large-cell carcinoma. In contrast, no patient with benign lung diseases had detectable serum IL-6 levels. Although Ershler *et al.* (1993) reported that the plasma levels of IL-6 rise with advancing age, there was no significant difference in age between any of the groups in the present study, as shown in Table I.

*Levels of CRP and fibrinogen in lung cancer patients with or without detectable serum IL-6 levels*

Since IL-6 is reported to stimulate hepatic protein synthesis *in vitro* (Ramadori *et al.*, 1988; Castell *et al.*, 1989), we examined hepatic protein levels in lung cancer patients and patients with benign lung diseases. Serum CRP and plasma fibrinogen were examined as hepatic proteins. As shown in Figure 2, patients with detectable serum IL-6 levels had significantly higher serum CRP levels ($65.9 \pm 2.9$ mg $l^{-1}$) than patients without detectable serum IL-6 levels ($18.2 \pm 0.9$ mg $l^{-1}$; $P<0.05$) and patients with benign lung diseases ($8.1 \pm 0.7$ mg $dl^{-1}$; $P<0.01$). Moreover, as shown in Figure 3, patients with detectable serum IL-6 levels had significantly higher plasma fibrinogen levels ($4.67 \pm 0.65$ g $l^{-1}$) than patients without detectable serum IL-6 levels ($3.80 \pm 0.38$ g $l^{-1}$; $P<0.05$) and patients with benign lung diseases ($3.21 \pm 0.70$ g $l^{-1}$; $P<0.05$).

**Figure 1** Serum IL-6 levels in patients with various types of lung cancer and with benign lung diseases. The lower limit of detection for the assay was 20 pg $ml^{-1}$.

**Table I** Clinical data of patients with benign lung diseases and patients with lung cancer

| | *No. of patients* | *Mean age (years) (range)* | *Sex* |
|---|---|---|---|
| Benign lung diseases | 20 | 60.0 (23–78) | 14M/6F |
| Lung cancer | 75 | 64.5 (44–85) | 62M/13F |
| Squamous cell carcinoma | 24 | 65.8 (45–85) | 23M/1F |
| Adenocarcinoma | 27 | 63.0 (44–83) | 18M/9F |
| Small-cell carcinoma | 20 | 66.2 (54–80) | 17M/3F |
| Large-cell carcinoma | 4 | 59.0 (45–68) | 4M/0F |

There was no significant difference in age between any groups.

*Serum albumin concentration in lung cancer patients with or without detectable serum IL-6 levels*

It has already been reported that serum albumin concentration can be considered as a marker of nutritional status in clinically ill patients (Blackburn *et al.*, 1977). To investigate nutritional status in patients with detectable serum IL-6 levels, we examined serum albumin concentration in lung cancer patients with or without detectable serum IL-6 levels as well as in patients with benign lung diseases. As shown in Table II, lung cancer patients with detectable serum IL-6 levels had a significantly lower serum albumin concentration than those without detectable serum IL-6 levels and patients with benign lung diseases ($P<0.05$).

*Blood platelet counts in lung cancer patients with or without serum IL-6 levels*

Since there are several reports demonstrating that IL-6 has thrombopoietic functions *in vitro* (Ikebuchi *et al.*, 1987; Ishibashi *et al.*, 1989), we studied the possible correlation between serum IL-6 concentrations and blood platelet counts. As shown in Figure 4, there was no significant difference in blood platelet counts between lung cancer patients with detectable ($26.5 \pm 0.51 \times 10^4$ $\mu l^{-1}$) or undetectable ($26.1 \pm 0.20 \times 10^4$ $\mu l^{-1}$) serum IL-6 levels or patients with benign lung diseases ($30.1 \pm 0.33 \times 10^4$ $\mu l^{-1}$).

**Figure 2** Mean serum CRP levels in patients with benign lung diseases and in lung cancer patients with undetectable and detectable serum IL-6 levels. NS, not significant.

**Figure 3** Mean plasma fibrinogen levels in patients with benign lung diseases and in lung cancer patients with undetectable and detectable serum IL-6 levels. NS, not significant.

**Figure 4** Mean blood platelet counts in patients with benign lung diseases and in lung cancer patients with undetectable and detectable serum IL-6 levels. No significant differences between the groups were seen.

**Table II** Serum albumin concentration in patients with benign lung diseases and in patients with lung cancer with or without detectable serum IL-6 levels

| | *Albumin* ($g\ dl^{-1}$) |
|---|---|
| Lung cancer patients | |
| with detectable IL-6 levels | 3.56 ± 0.02[a] |
| without detectable IL-6 levels | 3.81 ± 0.40[b] |
| Patients with benign lung diseases | 4.01 ± 0.02[b] |

[a]Mean ± s.e.m. [b]Statistically significant ($P<0.05$) compared with the data in lung cancer patients with detectable IL-6 levels.

**Table III** IL-6, CRP and fibrinogen levels in patients with various clinical stages of lung cancer

| *Clinical stage* | *IL-6* ($pg\ ml^{-1}$) | *CRP* ($mg\ l^{-1}$) | *Fib*[a] ($g\ l^{-1}$) |
|---|---|---|---|
| M-factor 0 | | | |
| Stage I ($n=7$) | 10.84 ± 2.70[b] | 0.9 ± 0.1 | 2.76 ± 0.1 |
| Stage II ($n=1$) | 0 | 0.5 | 3.1 |
| Stage IIIA ($n=10$) | 68.82 ± 9.86 | 32.4 ± 5.7 | 4.38 ± 0.3 |
| Stage IIIB ($n=17$) | 30.28 ± 2.72 | 53.8 ± 4.6 | 4.31 ± 0.1 |
| Total ($n=35$) | 36.54 ± 1.84 | 39.4 ± 1.9 | 4.00 ± 0.1 |
| M-factor 1 | | | |
| Stage IV ($n=40$) | 35.26 ± 2.05 | 32.4 ± 1.4 | 4.26 ± 0.1 |

[a]Fibrinogen. [b]Mean ± s.e.m.

At the same time, blood leucocyte counts were also examined in patients with these three groups. The absolute leucocyte counts were $9092 \pm 293\ \mu l^{-1}$ in lung cancer patients with detectable IL-6 levels, $7209 \pm 97\ \mu l^{-1}$ in lung cancer patients without detectable IL-6 levels and $6095 \pm 86\ \mu l^{-1}$ in patients with benign lung diseases. Lung cancer patients with detectable serum IL-6 levels had significantly higher blood leucocyte counts than patients with benign lung diseases ($P<0.05$).

### *Serum IL-6 and CRP levels and plasma fibrinogen levels in patients with various clinical stages of lung cancer*

We examined serum IL-6 and CRP levels and plasma fibrinogen levels in lung cancer patients with disease of different clinical stages. The data are shown in Table III. Although IL-6 and CRP levels were non-significantly lower in patients with stage I disease than in patients with stage IIIA, IIIB and IV disease, and plasma fibrinogen levels were significantly lower in patients with stage I disease than in those with stage IIIB and IV disease, there was no significant difference in serum IL-6 and CRP levels and plasma fibrinogen levels between patients with or without clinically demonstrated distant metastasis.

### Discussion

In the present study, we demonstrate that patients with lung cancer have elevated levels of IL-6, and that lung cancer patients with detectable serum IL-6 levels have significantly higher CRP and fibrinogen levels and lower albumin concentration than those without serum IL-6 levels and patients with benign lung diseases.

Elevated serum IL-6 levels have already been reported in elderly subjects (Ershler *et al.*, 1993) and in patients with various non-malignant diseases, including pneumonia (Moussa *et al.*, 1994), thermal injury (Nijsten *et al.*, 1987), sepsis (Helfgott *et al.*, 1989), human immunodeficiency virus (HIV)-related diseases (Breen *et al.*, 1990), rheumatoid arthritis (Houssiau *et al.*, 1988) and systemic lupus erythematosus (Spronk *et al.*, 1992). In the present study, there was no significant difference in age between patients with benign lung diseases and patients with lung cancer of various histological types, and patients with possible complications of benign diseases in which elevated serum IL-6 levels have been reported were strictly excluded. The mechanism responsible for the elevated levels of serum IL-6 in lung cancer patients remains unclear. It could be the result of abnormal production by tumour cells or a response of the immune system in the tumour-bearing state.

IL-6 is known to induce production of acute-phase proteins by hepatocytes (Ramadori *et al.*, 1988; Castell *et al.*, 1989). In our study, as observed previously by others in patients with renal cell carcinoma (Blay *et al.*, 1992), serum CRP levels in patients with detectable serum IL-6 levels were significantly higher than in those with undetectable levels. Moreover, as shown in Figure 3, plasma fibrinogen levels had the same correlation as CRP with serum IL-6 levels.

We have found that lung cancer patients with detectable serum IL-6 levels have lower serum albumin concentrations than those without detectable serum IL-6 levels. Dosquet *et al.* (1994) have already reported that renal cell carcinoma patients who experience weight loss have higher blood IL-6 levels than those without weight loss. As reported in animal models (Strassmann *et al.*, 1992), systemic IL-6 can be considered a mediator of malnutrition in lung cancer patients.

Megakaryocytopoiesis, resulting in the production and release of platelets, is regulated by various cytokines, including IL-1, IL-2, IL-3, IL-6, IL-7, IL-11, granulocyte colony-stimulating factor (CSF), macrophage CSF and granulocyte–macrophage CSF (Ogawa, 1993). Among these, IL-6 is reported to stimulate platelet production in animal models (Asano *et al.*, 1990; Hill *et al.*, 1990). Moreover, increased blood platelet counts were observed in a phase I study of subcutaneous administration of IL-6 (Wever *et al.*, 1993). Nevertheless, no significant thrombocytosis was observed in patients with detectable serum IL-6 levels in the present study. The reason for this discrepancy is unclear, but it is possible that the elevated IL-6 levels observed in patients with lung cancer are lower than required for stimulation of thrombopoiesis or that other additional cytokines necessary for thrombopoiesis were lacking in these patients.

There are several reports suggesting that IL-6 contributes to tumour progression directly and indirectly through inhibition of the anti-tumour response by host cells. In a murine model, IL-6 has been shown to enhance tumour progression, and local inhibition by IL-6 of the anti-tumour response by tumour-infiltrating lymphocytes has been suggested (Tanner and Tosato, 1991). Moreover, Dosquet *et al.* (1994) have reported that renal cell cancer patients with disseminated disease have higher blood IL-6 levels than those with undisseminated disease. On the other hand, detectable serum levels of IL-6 are less and less frequent with progression of B-cell chronic lymphocytic leukemia (Aderka *et al.*, 1993). In the present study, no statistical difference in serum IL-6 levels was observed in lung cancer patients with or without clinically demonstrated distant metastasis. Although the reason for this discrepancy is unclear, one possible explanation is the heterogeneity of the effect of IL-6 on growth of various tumour cells. An autocrine role for IL-6 in renal cell carcinoma (Miki *et al.*, 1989; Koo *et al.*, 1992) as well as multiple myeloma (Kawano *et al.*, 1988) and lymphoma (Yee *et al.*, 1989) has been reported, but Takizawa *et al.* (1993) reported that IL-6 may function as a growth-inhibiting factor in the proliferation of human lung cancer cell lines. Heterogeneity in the roles of IL-6 *in vivo* must be considered in various malignancies, and even in patients with lung cancer. Moreover, the presence of other soluble factors, such as soluble IL-6 receptor (Honda *et al.*, 1992), may affect the activity of IL-6 *in vivo*.

In summary, it is demonstrated in the present study that IL-6 may contribute to various reactions in patients with lung cancer. Further investigation is warranted to clarify the exact role of IL-6 in various malignancies.

**Acknowledgements**
This work was supported by a Grant-in-Aid for Cancer Research from the Ministry of Education, Science and Culture of Japan, and a grant from the Ministry of Health and Welfare of Japan. The authors thank the medical staff of the Third Department of Internal Medicine, Tokushima University Hospital, for kind support.

### References

ADERKA D, MAOR Y, NIVICK D, ENGELMANN H, KAHN Y, LEVO Y, WALLACH D AND REVEL M. (1993). Interleukin-6 inhibits the proliferation of B-chronic lymphocytic leukemia cells that is induced by tumour necrosis factor-α or -β. *Blood*, **81**, 2076–2084.

ASANO S, OKANO A, OZAWA K, NAKAHATA T, ISHIBASHI T, KOIKE K, KIMURA H, TANIOKA Y, SHIBUYA A, HIRANO T, KISHIMOTO T, TAKAKU F AND AKIYANA Y. (1990). *In vivo* effects of recombinant human interleukin-6 in primates: stimulated production of platelets. *Blood*, **75**, 1602–1605.

BLAY JY, NEGRIER S, COMBARET V, ATTALI S, GOILLOT E, MERROUCHE Y, MERCATELLO A, RAVAULT A, TOURANI JM, MOSKOVTCHENCKO JF, PHILIP T AND FAVROT M. (1992). Serum level of interleukin 6 as a prognosis factor in metastatic renal cell carcinoma. *Cancer Res.*, **52**, 3317–3322.

BLACKBURN GL, MAINI BS AND PIERCE JR EC. (1977). Nutrition in the clinically ill patient. *Anesthesiology*, **47**, 181–194.

BREEN EC, REZAI K, NAKAJIMA K, BEALL GN, MITSUYASU R, HIRANO T, KISHIMOTO T AND MARTINEZ-MAZA O. (1990). Infection with HIV is associated with elevated IL-6 levels and production. *J. Immunol.*, **144**, 480–484.

CASTELL JV, GOMEZ-LECHON MJ, DAVID M, ANDUS T, GEIGER T, TRULLENQUE R, FABRA R AND HEINRICH PC. (1989). Interleukin-6 is the major regulator of acute phase protein synthesis in adult human hepatocytes. *FEBS Lett.*, **242**, 237–239.

DOSQUET C, SCHAETZ A, FAUCHER C, LEPAGE E, WAUTIER JL, RICHARD F AND CABANE J. (1994). Tumour necrosis factor-α, interleukin-1β and interleukin-6 in patients with renal cell carcinoma. *Eur. J. Cancer*, **30A**, 162–167.

ERSHLER WB, SUN WH, BINKLEY N, GRAVENSTEIN S, VOLK MJ, KAMOSKE G, KLOPP RG, ROEXHER EB, DAYNES RA AND WEINDRUCH R. (1993). Interleukin-6 and aging: blood levels and mononuclear cell production increase with advancing age and *in vitro* production is modifiable by dietary restriction. *Lymphokine Cytokine Res.*, **12**, 225–230.

HELFGOTT DC, TATTER SB, SANTHANAM U, CLARICK RH, BHARDWAJ N, MAY LT AND SEHGAL PB. (1989). Multiple forms of IFN-$\beta_2$/IL-6 in serum and body fluids during acute bacterial infection. *J. Immunol.*, **142**, 948–953.

HILL RJ, WARREN MK AND LEVIN J. (1990). Stimulation of thrombopoiesis in mice by human recombinant interleukin 6. *J. Clin. Invest.*, **85**, 1242–1247.

HONDA M, YAMAMOTO S, CHENG M, YASUKAWA K, SUZUKI H, SAITO T, OSUGI Y, TOKUNAGA T AND KISHIMOTO T. (1992). Human soluble IL-6 receptor: its detection and enhanced release by HIV infection. *J. Immunol.*, **148**, 2175–2180.

HOUSSIAU FA, DEVOGELAER JP, VAN DAMME J, DE DEUXCHAISNES CN AND VAN SNICK J. (1988). Interleukin-6 in synovial fluid and serum of patients with rheumatoid arthritis and other inflammatory arthritides. *Arthritis Rheum.*, **31**, 784–788.

IKEBUCHI K, WONG GG, CLARK SC, IHLE JN, HIRAI Y AND OGAWA M. (1987). Interleukin-6 enhancement of interleukin-3 dependent proliferation of multipotential hematopoietic progenitors. *Proc. Natl Acad. Sci. USA*, **84**, 9035–9039.

ISHIBASHI T, KIMURA H, UCHIDA T, KARIYONE S, FRIESE P, BURSTEIN SA. (1989). Human interleukin 6 is a direct promoter of maturation of megakaryocytes *in vitro*. *Proc. Natl Acad. Sci. USA*, **86**, 5953–5957.

KAWANO M, HIRANO T, MATSUDA T, TAGA T, HORII Y, IWATO K, ASAOKU H, TANG B, TANABE O, TANAKA H, KURAMOTO A AND KISHIMOTO T. (1988). Autocrine generation and requirement of BSF-2/IL6 for human multiple myelomas. *Nature*, **332**, 83–85.

KISHIMOTO T. (1989). The biology of interleukin-6. *Blood*, **74**, 1–10.

KOO AS, ARMSTRONG C, BOCHNER B, SHIMABUKURO T, TSO CL, DEKERNION JB AND BELLDEGRUM A. (1992). Interleukin-6 and renal cell cancer: production, regulation and growth effects. *Cancer Immunol. Immunother.*, **35**, 97–105.

MATSUGUCHI T, OKAMURA S, KAWASAKI C, SHIMODA K, OMORI F, HAYASHI S, KIMURA N AND NIHO Y. (1991). Constitutive production of granulocyte colony-stimulating factor and interleukin-6 by a human lung cancer cell line, KSNY: gene amplification and increased mRNA stability. *Eur. J. Haematol.*, **47**, 128–133.

MEIR EV, SAWAMURA Y, DISERENS AC, HAMOU MF AND DE TRIBOLET N. (1990). Human glioblastoma cells release interleukin 6 *in vivo* and *in vitro*. *Cancer Res.*, **50**, 6683–6688.

MIKI S, IWANO M, MIKI Y, YAMAMOTO M, TANG B, YOKOKAWA K, SONODA T, HIRANO T AND KISHIMOTO T. (1989). IL-6 functions as an autocrine growth factor in renal cell carcinomas. *FEBS Lett.*, **250**, 607–610.

MIZUNO K, SONE S, ORINO E, NII A AND OGURA T. (1994). Autonomous expression of cytokine genes by human lung cancer cells and their paracrine regulation. *Jpn J. Cancer Res.*, **85**, 179–186.

MOUSSA K, MICHIE HJ, CREE IA, MCCAFFERTY AC, WINTER JH, DHILLON DP, STEPHENS S AND BROWN RA. (1994). Phagocyte function and cytokine production in community acquired pneumonia. *Thorax*, **49**, 107–111.

NIJSTEN MWN, DE GROOT R, DUIS HJT, KLASEN HJ, HACK CE AND AARDEN LA. (1987). Serum levels of interleukin-6 and acute phase responses. *Lancet*, **ii**, 921.

OGAWA M. (1993). Differentiation and proliferation of hematopoietic stem cells. *Blood*, **81**, 2844–2853.

RAMADORI G, DAMME JV, REIDER H AND ZUM BESCHENFELDE KM. (1988). Interleukin 6, the third mediator of acute-phase reaction, modulates hepatic protein synthesis in human and mouse: comparison with interleukin 1 and tumor necrosis factor. *Eur. J. Immunol.*, **18**, 1259–1264.

RAWLE FC, SHIELDS J, SMITH SH, LIESCU V, MERKENSCHLAGER M, BEVERLY PCL AND CALLARD RE. (1989). B cell growth and differentiation induced by supernatants of transformed epithelial cell lines. *Eur. J. Immunol.*, **16**, 1017–1019.

SPRONK PE, TER BORG EJ, LIMBERG PC AND KALLENBERG CG. (1992). Plasma concentration of IL-6 in systemic lupus erythematosus; an indicator of disease activity? *Clin. Exp. Immunol.*, **90**, 106–110.

STRASSMANN G, FONG M, KENNEY JS AND JACOB CO. (1992). Evidence for the involvement of IL-6 in experimental cancer cachexia. *J. Clin. Invest.*, **89**, 1681–1684.

TAKIZAWA H, OHTOSHI T, OHTA K, YAMASHITA N, HIROHATA S, HIRAI K, HIRAMATSU K AND ITO K. (1993). Growth inhibition of human lung cancer cell lines by interleukin 6 *in vitro*: a possible role in tumor growth via an autocrine mechanism. *Cancer Res.*, **53**, 4175–4181.

TANNER J AND TOSATO G. (1991). Impairment of natural killer functions by interleukin 6 increased lymphoblastoid cell tumorigenicity in athymic mice. *J. Clin. Invest.*, **88**, 239–247.

UICC. (1987). *TNM Classification of Malignant Tumours*. 4th edn. UICC: Geneva.

WATSON JM, SENSINTAFFAR JL, BEREK JS AND MARTINEZ-MAZA O. (1990). Constitutive production of interleukin 6 by ovarian cancer cell lines and by primary ovarian tumor cultures. *Cancer Res.*, **50**, 6959–6965.

WEVER J, YANG JC, TOPALIAN SL, PARKINSON DR, SCHWARTZENTRUBER DS, ETTINGHAUSEN SE, GUNN H, MIXON A, KIM H, COLE D, LEVIN R AND ROSENBERG SA. (1993). Phase I trial of subcutaneous interleukin-6 in patients with advanced malignancies. *J. Clin. Oncol.*, **11**, 499–506.

YANAGAWA H, SONE S, MUNEKATA M, ATAGI S, NII A. AND OGURA T. (1992). IL-6 in malignant pleural effusions and its augmentation by intrapleural instillation of IL-2. *Clin. Exp. Immunol.*, **88**, 207–212.

YEE C, BIONDI A, WANG XH, ISCOVE NN, DESOUSA J, AARDEN LA, WONG GG, CLARK SC, MESSNER HA AND MINDEN MD. (1989). A possible autocrine role for interleukin-6 in two lymphoma cell lines. *Blood*, **74**, 798–804.